# Risk factors and surgical prognosis in patients with aortic valve involvement caused by Takayasu arteritis

**DOI:** 10.1186/s13075-022-02788-9

**Published:** 2022-05-07

**Authors:** Xuemei Shi, Juan Du, Taotao Li, Na Gao, Wei Fang, Suwei Chen, Zhiyu Qiao, Chengnan Li, Junming Zhu, Lili Pan

**Affiliations:** 1 Department of Rheumatology and Immunology, Beijing Anzhen Hospital, Capital Medical University, No. 2 Anzhen Road, Chaoyang District, Beijing, 100029 China; 2 Department of Pathology, Beijing Anzhen Hospital, Capital Medical University, Beijing, 100029 China; 3 Department of Cardiovascular Surgery, Beijing Aortic Disease Centre, Beijing Anzhen Hospital, Capital Medical University, Beijing Institute of Heart Lung and Blood Vessel Diseases, Beijing Engineering Research Centre for Vascular Prostheses, No. 2 Anzhen Road, Chaoyang District, Beijing, 100029 China

**Keywords:** Takayasu arteritis, Aortic valve, Surgical treatment, Anti-inflammatory therapy

## Abstract

**Objective:**

Aortic valve involvement is not uncommon in patients with Takayasu arteritis (TAK) and leading to poor prognosis. The aim of our study was to explore the risk factors of aortic valve involvement and to evaluate the prognosis in TAK patients with aortic valve involvement.

**Method:**

In this retrospective study, 172 TAK patients were divided into groups with or without aortic valve involvement to identify the risk factors. Patients who underwent aortic valve surgical treatment were followed up to assess cumulative incidence of postoperative adverse events.

**Results:**

A total of 92 TAK patients (53.49%) had aortic valvular lesion. The infiltration of inflammatory cells was found in surgical specimens of aortic valve. Numano type IIb, elevated high-sensitivity C-reactive protein (hs-CRP) level, and dilation of ascending aorta and aortic root were statistically associated with aortic valvular lesion in TAK patients (OR [95%CI] 6.853 [1.685–27.875], *p*=0.007; 4.896 [1.646–14.561], *p*=0.004; 4.509 [1.517–13.403], *p*=0.007; 9.340 [2.188–39.875], *p*=0.003). The 1-, 5-, and 7-year cumulative incidence of postoperative adverse events were 14.7%, 14.7%, and 31.8%, respectively. Surgical methods (*p*=0.024, hazard ratio (HR) 0.082) and postoperatively anti-inflammatory therapy (*p*=0.036, HR 0.144) were identified as potential predictors of postoperative adverse events.

**Conclusions:**

Regularly echocardiogram screening is suggested in patients with Numano type IIb and aggressive treatment should be performed early in TAK patients. As for TAK patients with aortic valve surgery, aortic root replacement seems to be the preferred option and regular anti-inflammatory therapy may reduce the occurrence of adverse events of them.

**Supplementary Information:**

The online version contains supplementary material available at 10.1186/s13075-022-02788-9.

## Introduction

Takayasu arteritis (TAK) is a rare chronic systemic vasculitis that mainly affects the aorta and its major branches. The etiology and pathogenesis of TAK have not been fully clarified. Most of the TAK cases occur in Asia and the Middle East, with young female patients mainly affected and the onset age is usually between 10 and 40 years [1]. TAK is characterized by granulomatous full-thickness arteritis in the involved arteries, which can lead to lumen stenosis or even occlusion, and in a few cases, arterial dilation or aneurysm, resulting in ischemia of corresponding blood supplying organs.

TAK can also involve cardiac valves. Mwipatayi et al. reported that 22% (61/272) of TAK patients had cardiac valvular lesion [2]. In Chinese patients with TAK, valvular abnormalities were found in 34.9% (373/1069) to 64.08% (66/103) patients [3–6]. In addition, the most common valve involved in TAK is aortic valve, and the most common valvular lesion caused by TAK is valvular insufficiency. Therefore, aortic regurgitation (AR) is a relatively common and important complication observed in TAK patients, which is considered to be caused by the inflammation of aortic root and valve [7]. The incidence of AR in patients with TAK has been reported as 33.2% (455/1372) in Japan [8], 21.7% (13/60) in Italy [9], 18.1% (29/160) in Korea [10], and 20.4% (84/411) [3] to 39.8% (41/103) [6] in China. AR can further induce congestive heart failure or arrhythmia, both of which are considered one of the main causes of death in TAK patients [11].

Thus, we retrospectively reviewed the medical records of TAK patients admitted to our hospital and collected follow-up data to investigate the clinical characteristics of aortic valve involvement in these patients, identify the associated risk factors and explore the long-term clinical outcomes in patients with aortic valve surgery and potential prognostic predictors.

## Materials and methods

### Participants

One hundred seventy-two patients admitted to Beijing Anzhen Hospital between January 2014 and February 2021 with diagnosis of Takayasu’s disease confirmed by the criteria for classifying TAK developed by the American College of Rheumatology in 1990 [12]. The exclusion criteria were patients without echocardiography during hospitalization or in pre-aortic-valve-operation and those with congenital heart disease or other autoimmune diseases. The study was approved by the Ethics Committee of Beijing Anzhen Hospital (approval number: 2022051X) and conformed to the ethical guidelines of the Helsinki Declaration and its amendments. Considering the retrospective design of this study, the requirement of written informed consent was exempted.

### Clinical data collection

For patients with multiple hospitalizations, we only analyzed records of their first hospitalization. We retrospectively reviewed the patients’ demographic date, clinical manifestations, laboratory parameters, imaging, and pathological examinations. The angiographic type of TAK was classified according to the criteria established by Hata and Numano [13]. And the involvement of coronary and pulmonary artery should be indicated as C or P, respectively.

The National Institutes of Health (NIH) criteria [14], the Indian Takayasu’s Arteritis Activity Score (ITAS2010) [15], and ITAS with acute-phase reactants (ITAS-A) [16] were used for assessment of disease activity.

All laboratory parameters are routine examination items of clinical laboratory department in our hospital. The level of erythrocyte sedimentation rate (ESR) >20mm/h for female and >15mm/h for male, high-sensitivity C-reactive protein (hs-CRP) >5mg/l, tumor necrosis factor alpha (TNF-α) >8.1pg/ml, interleukin 6 (IL-6) >5.9pg/ml, immunoglobulin A (IgA) >4.2g/l, immunoglobulin G (IgG) >17.4g/l, immunoglobulin M (IgM) >2.8g/l, complement proteins C3 >1.4g/l, and C4 >0.4g/l were defined as elevated level.

### Evaluation of aortic valve involvement

Aortic valve involvement was defined as valve thickening, calcification, prolapse, contracture, stenosis, and insufficiency (evaluated with B-mode and color Doppler echocardiography). Mild, moderate, and severe aortic valve stenosis and insufficiency were defined according to the 2014 AHA/ACC Guideline for the Management of Patients with Valvular Heart Disease [17]. According to Recommendations for Cardiac Chamber Quantification by Echocardiography in Adults [18], with B-mode transthoracic echocardiography, aortic root dilatation at the sinuses of Valsalva is defined as an aortic root diameter above 37mm for male and 33mm for female, while the diameter of ascending aorta >34mm for male and >31mm for female was defined as expansion.

### Pathological staining of aortic valve tissue

Twenty-five pathological specimens of aortic valve and 18 cases of aortic wall intraoperatively from 32 TAK patients were studied. The specimens for histological study were taken intraoperatively and fixed in 4% neutral formalin for 24 h, embedded in paraffin, and sectioned at 7-μm thickness. Hematoxylin-eosin stain was evaluated to demonstrate infiltration of inflammatory cells, fibrous hyperplasia, and areas of hyaline degeneration and mucoid degeneration.

### Follow-up and outcome

Thirty-four patients who underwent aortic valve surgical treatment were followed up from the date of surgery to January 2022. The follow-up data, including clinical symptoms, laboratory tests, echocardiography, and medication, were obtained by either telephone interview or re-hospitalization records.

Outcomes, comprising all-cause death, paravalvular leak, anastomotic leakage, detachment of prosthetic valve, ascending aortic aneurysm, anastomotic aneurysm, recurrent sever aortic regurgitation, non-fatal cardiac events, and neurologic events, were defined as end points according to the Updated Endpoint Definitions for Aortic Valve Clinical Research [19].

### Statistical analysis

Continuous variables are described as mean ± SD for normally distributed date and as median (Q1, Q3) for non-normally distributed date. Classification variables are presented as count (percentage). Different statistical methods, including the independent *t*-test, chi-square tests, Fisher’s exact test, or the Mann–Whitney U test, were used to examine differences, as appropriate. Binary logistic regression was used to identify the independent risk factors associated with aortic valve involvement. Cumulative adverse event rates were determined by Kaplan–Meier curve analysis, and potential predictors of prognosis were identified by Cox proportional hazard regression analysis. All statistical tests were two-tailed, and statistical significance was set at *P* valves<0.05. The statistical analysis was performed with SPSS software (version 25.0; IBM Corp., Armonk, NY, USA), and the figures were created by GraphPad Prism 9 (GraphPad Software, San Diego, CA, USA).

## Results

### Clinical manifestations at baseline

Among the 172 TAK patients, 92 cases (53.49%) had aortic valve involvement, with a female/male ratio of 7.36:1 (81/11). Patients with aortic valve involvement had a higher age of symptom onset (30.0 vs. 26.5 years, *P*=0.030) than patients without aortic valve involvement. Moreover, we compared the history of TAK patients and found no significant difference in terms of hypertension, hyperlipidemia, diabetes mellitus, and coronary heart disease between the two groups except smoking history.

There were significantly different in chest tightness (*P*=0.001), shortness of breath (*P*=0.001), and intermittent claudication (*P*=0.022) between the two groups, which were no significant differences in other symptoms (Table 1). We also found no significant difference in terms of sinus tachycardia, sinus bradycardia, premature beats, atrial fibrillation, and atrioventricular block between the two groups (Table 2).

**Table 1:** Clinical characteristic of TAK patients with or without aortic valve involvement

Variable	Total (*n*=172)	Aortic valve involvement (*n*=92)	Non-aortic valve involvement (*n*=80)	*P*
Age at symptom onset, years	28.00 (23.00, 38.00)	30.00 (23.00, 41.75)	26.50 (20.00, 36.00)	0.030
Duration of disease, months	48.00 (12.00, 144.00)	60.00 (12.00,165.00)	36.00 (8.25, 120.00)	0.160
Female, *n* (%)	147 (85.5)	81 (88.0)	66 (82.5)	0.304
History, *n* (%)				
Smoking	21 (12.2)	6 (6.5)	15 (18.8)	0.015
Hypertension	52 (30.2)	28 (30.4)	24 (30.0)	0.951
Hyperlipidemia	17 (9.9)	9 (9.8)	8 (10.0)	0.962
Diabetes mellitus	8 (4.7)	2(2.2)	6 (7.5)	0.197
Coronary artery disease	15 (8.7)	11 (12.0)	4 (5.0)	0.107
Medication	59 (34.3)	36 (39.1)	23 (28.7)	0.153
Glucocorticoid	53 (31.5)	32 (36.4)	21 (26.3)	0.159
Immunosuppressant	42 (25.0)	23 (26.1)	19 (23.8)	0.721
Clinical symptoms, *n* (%)				
Chest tightness	58 (33.7)	41 (44.6)	17 (21.3)	0.001
Chest pain	34 (19.8)	21 (22.8)	13 (16.3)	0.280
Palpitation	24 (14.0)	16 (17.4)	8 (10.0)	0.163
Shortness of breath	19 (11.0)	17 (18.5)	2 (2.5)	0.001
Hemoptysis	5 (2.9)	2 (2.2)	3 (3.8)	0.874
Dizziness	71 (41.3)	39 (42.4)	32 (40.0)	0.751
Amaurosis	6 (3.5)	4 (4.3)	2 (2.5)	0.809
Consciousness	8 (4.7)	3 (3.3)	5 (6.3)	0.572
Fever	21 (12.2)	13 (14.1)	8 (10.0)	0.409
Fatigue	72 (41.9)	38 (41.3)	34 (42.5)	0.874
Headache	27 (15.7)	12 (13.0)	15 (18.8)	0.305
Carotidynia	20 (11.6)	8 (8.7)	12 (15.0)	0.198
Limb pain	19 (11.0)	12 (13.0)	7 (8.8)	0.370
Intermittent claudication	27 (15.7)	9 (9.8)	18 (22.5)	0.022
Weak pulse or pulselessness	39 (22.7)	21 (22.8)	18 (22.5)	0.959
Upper limb blood pressure asymmetry	53 (30.8)	25 (27.2)	28 (35.0)	0.268
Type^a^, *n* (%)				
I	23 (15.8)	7 (10.4)	16 (20.3)	0.105
IIa	7 (4.8)	5 (7.5)	2 (2.5)	0.317
IIb	22 (15.1)	16 (23.9)	6 (7.6)	0.006
III	7 (4.8)	1 (1.5)	6 (7.6)	0.183
IV	4 (2.7)	0 (0)	4 (5.1)	0.174
V	83 (56.8)	38 (56.7)	45 (57.0)	0.976
C	35 (24.0)	21 (31.3)	14 (17.7)	0.055
P	26 (17.8)	15 (22.4)	11 (13.9)	0.183

^a^Type is defined according to the distribution of the involved artery; Type is divided according to Hata and Numano’s criteria; C, indicates any lesion with coronary artery involvement; P, indicates any lesion with pulmonary artery involvement

**Table 2:** Arrhythmia and echocardiographic parameters in TAK patients with or without aortic valve involvement

Variable	Total (*n*=172)	Aortic valve involvement (*n*=92)	Non-aortic valve involvement (*n*=80)	*P*
Arrhythmia, *n* (%)				
Sinus tachycardia	19 (11.5)	11 (12.5)	8 (10.4)	0.672
Sinus bradycardia	6 (3.6)	5 (5.6)	1 (1.3)	0.285
Premature beats	6 (3.6)	2 (2.2)	4 (5.2)	0.550
Atrial fibrillation	4 (2.4)	2 (2.2)	2 (2.6)	1.000
Atrioventricular block	4 (2.4)	2 (2.2)	2 (2.6)	1.000
Echocardiography				
EF, %	63.00 (58.00, 67.25)	61.45 (56.00, 66.75)	65.50 (60.00, 69.00)	0.002
LVEDD, mm	47.00 (43.75, 54.00)	51.50 (46.13, 58.00)	44.00 (42.00, 47.48)	<0.001
LVESD, mm	30.00 (27.00, 36.00)	33.00 (29.00, 40.00)	27.50 (26.85, 30.25)	<0.001
LAD>38mm	57 (34.3)	45 (51.7)	12 (15.2)	<0.001
Aortic root diameter, mm	31.00 (28.00, 35.25)	34.00 (30.00, 39.00)	30.00 (27.00, 32.00)	<0.001
Dilated aortic root diameter, *n* (%)	47 (29.0)	43 (50.6)	4 (5.2)	<0.001
Ascending aorta diameter, mm	34.00 (29.50, 39.00)	36.00 (34.00, 42.00)	31.15 (28.00, 34.00)	<0.001
Dilated ascending aorta diameter, *n* (%)	101 (66.0)	72 (86.7)	29 (41.4)	<0.001
Mitral valve involvement, *n* (%)	81 (47.6)	62 (68.1)	19 (24.1)	<0.001
Tricuspid valve involvement, *n* (%)	63 (36.8)	41 (45.1)	22 (27.5)	0.018
Pulmonary valve involvement, *n* (%)	3 (1.8)	2 (2.3)	1 (1.3)	1.000

*EF%* cardiac ejection fraction, *LVEDD* left ventricular end-diastolic diameter, *LVESD* left ventricular end-systolic diameter, *LAD* left atrial diameter

As for the medication of TAK patients before their first admission, 51 patients (30.7%) were treated with glucocorticoids and 41 (24.7%) were treated with immunosuppressants, but there was no statistically significant difference in drug treatments between the two groups (Supplementary Table 1).

### Characteristics of aortic valvular lesion evaluated by Doppler echocardiography

Ninety-two TAK patients (53.49%) had aortic valvular lesion. Aortic valvular insufficiency (88/92, 95.7%) was the most common aortic valvular lesion, and more than half of them (51/92, 55.4%) had moderate to severe aortic regurgitation. There were two patients (2.2%) with aortic stenosis, and one patient had both moderate aortic stenosis and mild regurgitation. Thickening of aortic valve was the most common structural damage (23/92, 25.0%). Five (5.4%) patients had aortic valvular calcification, and 3 (3.2%) had aortic valvular prolapse. Three patients had only aortic valvular thickening and calcification, but no valve stenosis or insufficiency.

Detailed echocardiographic parameters are presented in Table 2. Compared with patients without aortic valve involvement, people had larger left ventricular end-diastolic diameter (LVEDD) (51.5 vs. 44.0mm, *P*<0.001) and lower ejection fraction (EF) (61.5 vs. 65.5%, *P*=0.002) among patients with aortic valvular lesion. Furthermore, there was statistically significant difference in diameter of the aortic root (34.0 vs. 30.0mm, *P*<0.001) and ascending aorta (36.0 vs. 31.15mm, *P*<0.001) between the two groups.

Among the 92 TAK patients with aortic valve lesion, 62 (68.1%) had mitral valvular involvement, 41 (45.1%) tricuspid valvular involvement, and 2 (2.3%) pulmonary valvular involvement. There was significant difference in proportion of mitral and tricuspid valvular involvement between the two groups.

### Numano type, laboratory parameters, and disease activity of TAK

We found that Numano type V was the most common type. Among patients with aortic valve involvement, the proportion of them with Numano type IIb was significantly higher than that in the non-aortic valve lesion group (23.9% vs. 7.6%, *P*=0.006). There was no significant difference in the frequency of type I, IIa, III, IV, and V between the two groups. We also analyzed the involvement of coronary artery and pulmonary artery involvement of TAK patients and found no significant difference between the two groups (Table 1).

**Fig. 1:**
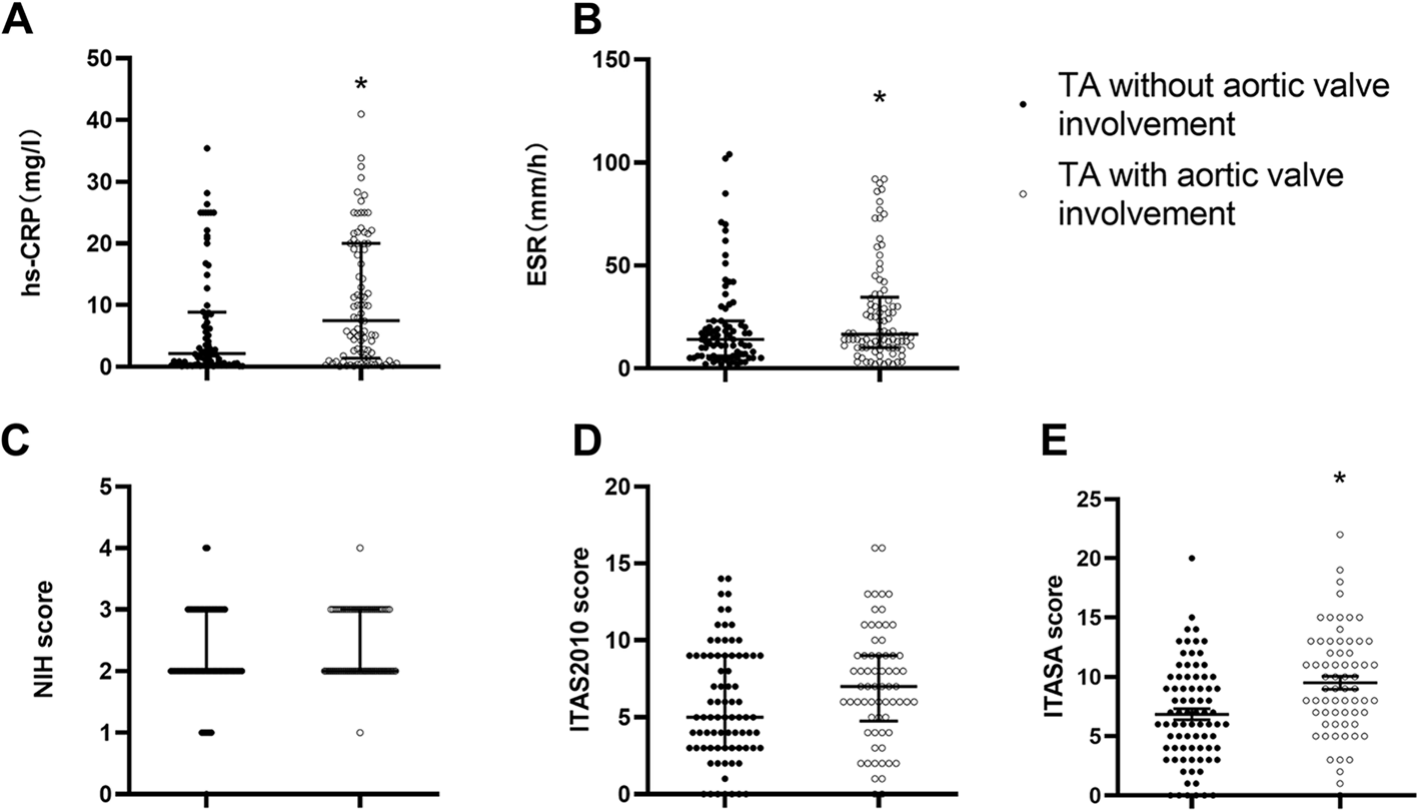
Disease activity in TA patients with or without aortic valve involvement. The proportion of elevated ESR and elevated hs-CRP, and ITAS-A score in the aortic valve involvement group were significantly higher than in the non-aortic valve involvement group (* *P*<0.05). ESR, erythrocyte sedimentation rate; hs-CRP, high-sensitivity C-reactive protein

The proportion of elevated ESR and hs-CRP, and ITAS-A score in the aortic valve involvement group were significantly higher than that in the non-aortic valve involvement group [45.6% vs. 30.4%, *P*=0.043; 60.2% vs. 36.4%, *P*=0.002; 9.0 (6.8,13.0) vs. 6.0 (4.0,10.0), *P*<0.001] (Fig. 1).

Patients had higher proportion of elevated IgG and higher level of brain natriuretic peptide (BNP) and cardiac troponin I (cTnI) in the aortic valve involvement group than in the non-aortic valve involvement group (26.5% vs. 10.3%, *P*=0.011; 189.0 vs. 34.5pg/ml, *P*=0.004; 0.01 vs. 0.00ng/ml, *P*=0.001). The level of white blood cell (WBC), red blood cell (RBC), platelet, alanine aminotransferase, creatinine, IL-6, TNF-α, IgA, IgM, C3, and C4 were not different among the two groups (Supplementary Table 2).

### Risk factors of aortic valve involvement in TAK patients

**Fig. 2:**
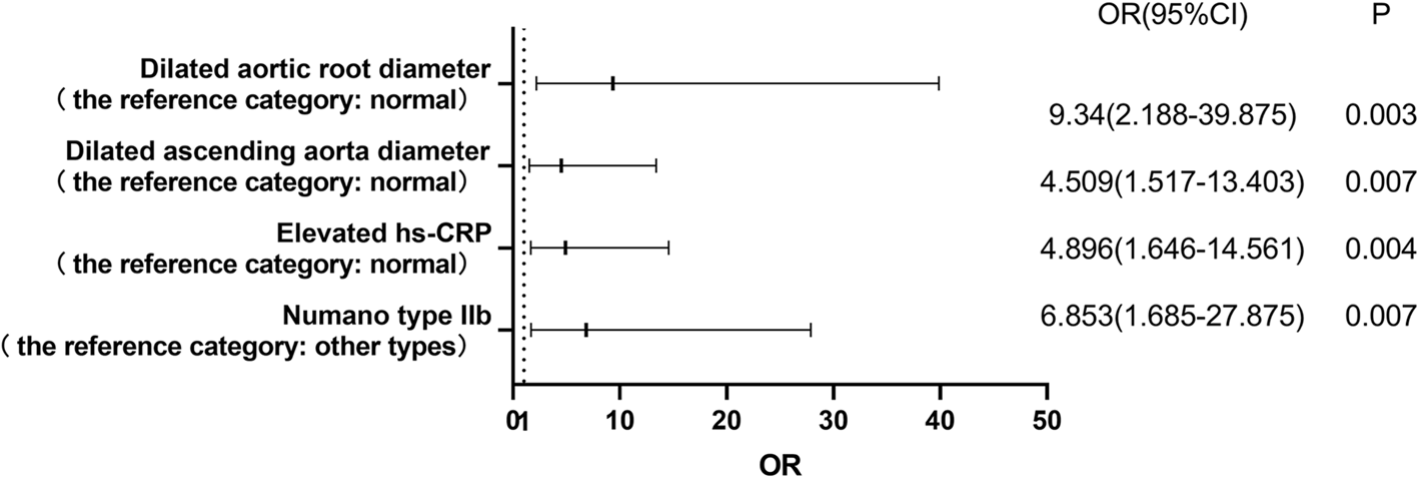
Logistics regression analysis of risk factors in TA patients with aortic valve involvement. Numano type IIb, hs-CRP level, ascending aorta diameter, and aortic root diameter are significantly related to aortic valve involvement in TA patients

We included the symptom onset age, duration of disease, gender, smoking history, ESR, hs-CRP, IgG, Numano type, aortic root diameter, and ascending aorta diameter in the logistic regression analysis of risk factors of aortic valve involvement in TAK patients. The Numano type IIb, elevated hs-CRP level, and dilation of ascending aorta and aortic root were found to be statistically related to aortic valvular lesion in TAK patients (OR [95%CI] 6.853 [1.685–27.875], *p*=0.007; 4.896 [1.646–14.561], *p*=0.004; 4.509 [1.517–13.403], *p*=0.007; 9.340 [2.188–39.875], *p*=0.003) (Fig. 2).

### Pathological features of aortic valve lesions in TAK patients

**Fig. 3:**
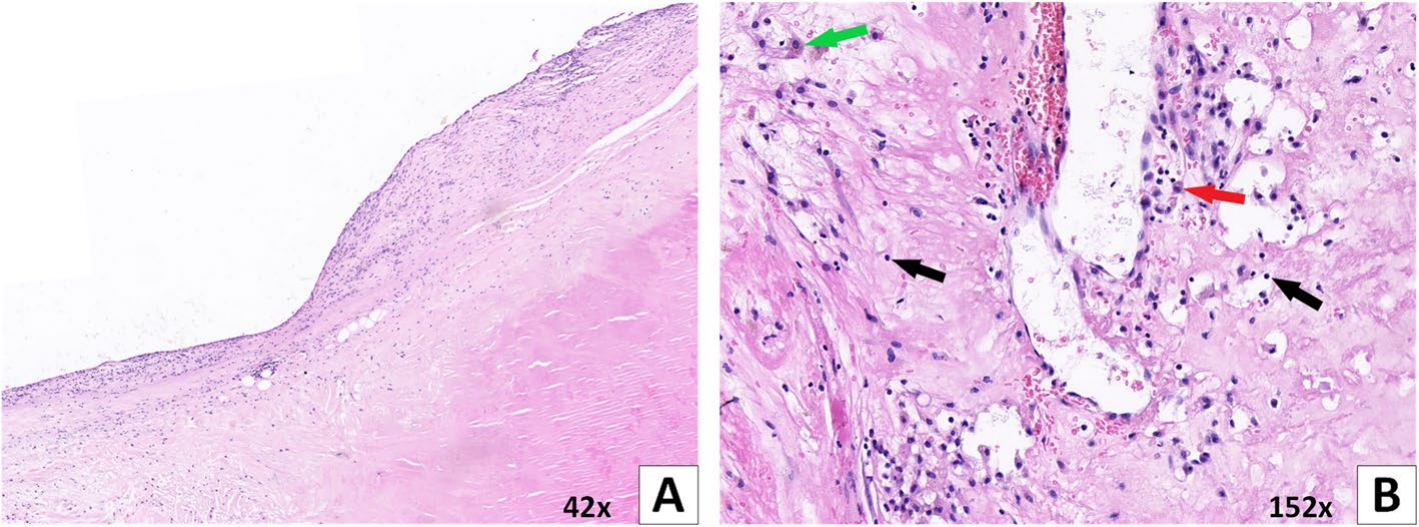
Surgical specimen of the aortic valve (H&E). **A**, **B** Aortic valve tissue from the same 41-year-old male TA patient, diagnosed after aortic valve replacement due to moderate aortic valve stenosis and mild regurgitation. **A** The markedly lymphocytic infiltration and thickening of aortic valve edge was observed (H&E 42×). **B** Chronic inflammatory cells infiltrating was present, such as lymphocyte (black arrow), plasmacyte (red arrow), and macrophage (green arrow) (H&E 152×)

Thirty-two TAK patients with aortic regurgitation or stenosis were surgically treated at Beijing Anzhen Hospital during the 7-year period, while 2 patients at other hospitals. Twenty-five pathology reports of the resected aortic valve and 18 cases of the resected aortic wall intraoperatively were recorded. With regard to aortic valves, 24 (96.0%) patients had valvular fibrosis, 23 (92.0%) mucoid degeneration, and 19 (76.0%) glassy degeneration. Two patients (8.0%) were found to have infiltration of inflammatory cells in aortic valve (Fig. 3). Furthermore, we also found lymphocytic infiltration of aortic wall in 7 patients (38.9%), with infiltration of plasma cells in 3 of them, which mainly occurred in the tunica media and tunica adventitia. Thickening, fibrous hyperplasia, and hyaline degeneration of intima and adventitia of aorta were found in the vast majority of TAK patients. As for the media of aorta, the most common injuries were rupture and disorder of elastic fibers and degeneration and disorder of smooth muscle cells.

### Prognosis analysis

**Fig. 4:**
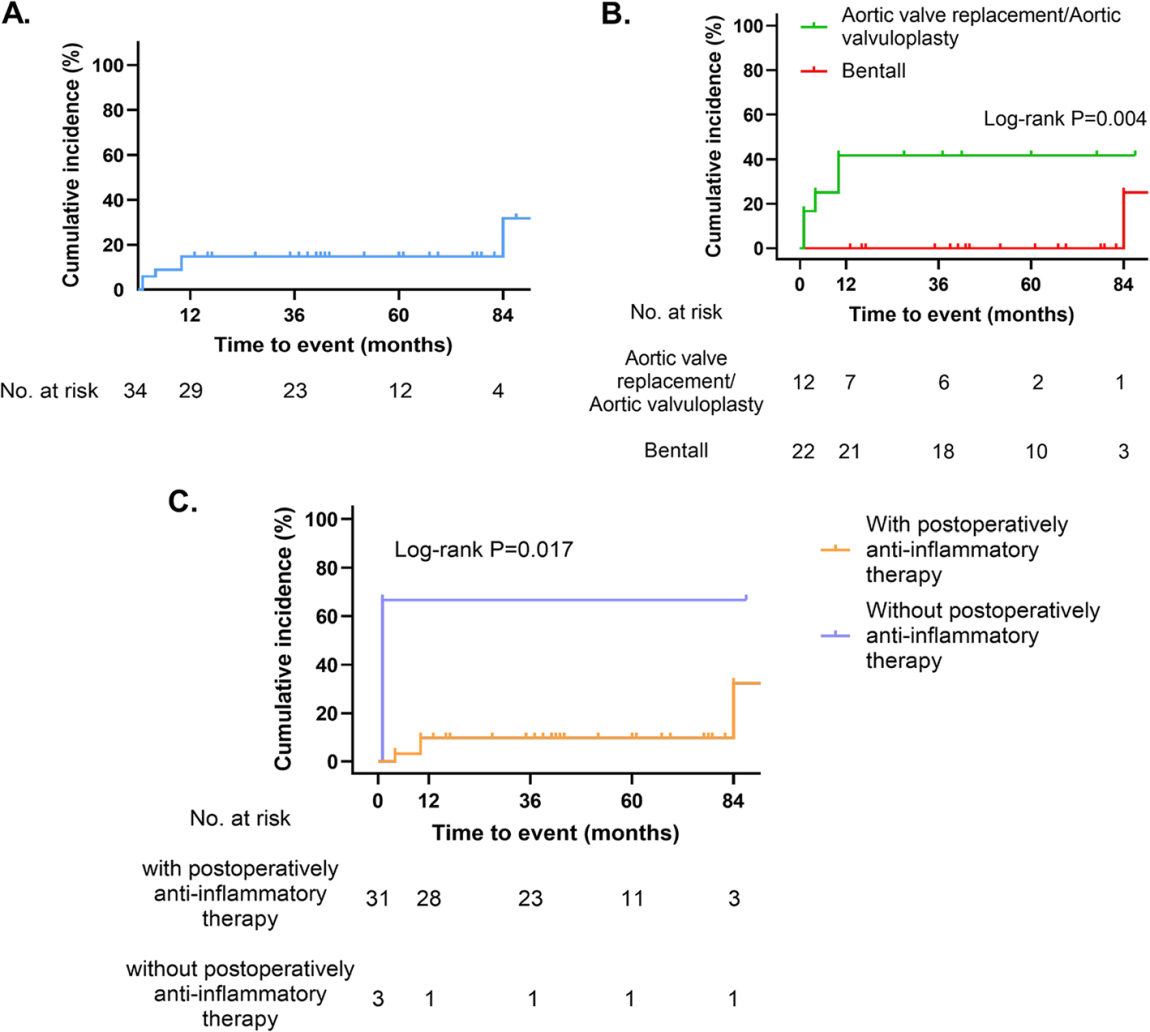
Cumulative incidence of postoperative adverse events in TA patients with aortic valve lesion. **A** Cumulative incidence of postoperative adverse events in 34 TA patients who underwent aortic valve surgical treatment. Kaplan–Meier analysis of postoperative adverse events according to **B** different surgical methods and **C** postoperatively anti-inflammatory therapy

The mean follow-up was 46.9 months and the median follow-up was 42.5 months. Aortic valve replacement (AVR) was performed in 11 patients, aortic valvuloplasty was performed in one patient, and aortic root replacement (also known as Bentall) was performed in 22 patients. Six end-point events were observed, including two deaths, one paravalvular leak, one anastomotic leakage, one ascending aortic pseudoaneurysm, and one recurrent sever aortic regurgitation. The 1-, 5-, and 7-year cumulative incidence of adverse events were 14.7%, 14.7%, and 31.8%, respectively (Fig. 4A).

The univariate Cox regression analysis showed that surgical methods (*p*=0.024, hazard ratio (HR) 0.082) and postoperatively anti-inflammatory therapy (*p*=0.036, HR 0.144) may be potential predictors of postoperative adverse events in TAK patients (Supplementary Table 3). Surgical methods can be divided into two groups, one is isolated aortic valve surgery, including AVR and aortic valvuloplasty, and the other is aortic root replacement. Assignment to the different surgery group was a shared decision between surgeons and patients based on the patient’s condition. Patients who regularly used glucocorticoid, immunosuppressants, or biological agents singly or jointly were included in the postoperative anti-inflammatory treatment group. According to the Kaplan–Meier curve analysis, there were significant differences between subgroups (Fig. 4B, C).

A total of 3 patients did not receive postoperative anti-inflammatory therapy, and two of them developed adverse events early. One patient, a 44-year-old woman, developed anastomotic leakage 1 month after aortic valve replacement and ascending aorta replacement. The other patient was a 41-year-old male who developed a pseudoaneurysm and perforation of the ascending aorta (near the previous surgical incision) 2 months after aortic valve replacement. Two of them did not begin regular anti-inflammatory therapy until an adverse event occurred, while another patient who did not have an adverse event had irregular anti-inflammatory therapy years after aortic valve surgery due to poor compliance.

Due to the lack of end-point events, no multivariate Cox regression analysis was performed.

## Discussion

In our study, we documented that 53.5% of TAK patients had aortic valvular involvement. Aortic valvular insufficiency was the most common aortic valvular lesion, and Numano type V was the most common type, followed by Numano type IIb. Inflammatory cells infiltrating was found in our pathological specimens of the resected aortic valve. We first found that dilation of ascending aorta and aortic root, Numano type IIb, and elevated hs-CRP level were significantly associated with aortic valve involvement in TAK patients. Surgical methods and postoperatively anti-inflammatory therapy were identified as potential predictors of postoperative adverse events.

The incidence of AR in TAK patients in our research was 51.2% (88/172), which was higher than that reported in previous studies, showing an incidence rate ranging from 13 to 44% [20–23]. This may be partly because our hospital is a general hospital with cardiovascular diseases. And more than half of them (58.0%, 51/88) were moderate to severe AR, indicating that the damage of aortic valve is serious caused by TAK, which reminded clinicians to early intervention. It is believed that the most common cardiac manifestation of TAK is AR, which could lead to myocardial remodeling and left ventricle dysfunction [24], and is considered a significant risk factor for mortality in patients with TAK [20, 25–27].

Our study showed that patients with aortic valve involvement had a higher age of symptom onset, which was similar to the findings of previous researches about TAK [5, 6]. The older the patient’s onset age, the more easily various risk factors such as physical and hemodynamic factors may cause damage of aortic valve, combining with the inflammatory damage of vasculitis itself.

Previous researches indicated that Numano type V was the most common angiographic involvement pattern [4, 5, 10, 28], which was consistent with our study. We also found that there was significant difference in the frequency of type IIb between the two groups. The risk of aortic valve involvement in TAK patients with Numano type IIb was 6.9-fold higher than that of patients with other types. In clinical practice, it is recommended that patients with type IIb should regularly perform the echocardiogram screening, so as to detect aortic valve lesion early, guide timely intervention, and avoid further valvular damage.

As for the TAK disease activity indicators, there was significant difference in the level of ESR and hs-CRP and ITAS-A score between the two groups. Disease activity played an important role in cardiovascular manifestations of TAK, and the incidence of aortic valve regurgitation was higher in the active TAK group with a statistically significant difference [10]. The prognosis of TAK depends mainly on the disease activity and complications with cardiac involvement [29]. We found that the risk of aortic valve lesion in TAK patients with elevated hs-CRP level was 4.9-fold higher than in patients with normal hs-CRP, suggesting inflammation is associated with aortic valve lesion [30]. Hs-CRP level was independently associated with the presence of cardiovascular events in TAK patients [31], and elevated hs-CRP could predict cardiovascular events in TAK patients with coronary artery disease [32]. A multicenter study found that elevated CRP level was associated with relapse of TAK [28]. Furthermore, direct infiltration of inflammatory cells, like lymphocyte and plasma cells, into aortic valve were firstly observed in the pathological slices of our study, while other studies showed no evidence of inflammation in aortic valve [5, 20, 33]. Therefore, inflammation and TAK disease activation may directly cause the aortic valvular dysfunction through damaging aortic valvular structure, which is confirmed in our research, finding the valvular fibrosis, mucoid degeneration, and glassy degeneration in pathological specimen of aortic valve.

In addition, patients had higher level of IgG in the aortic valve involvement group. One possible explanation was that a previous study suggested that serum IgG drives endothelial remodeling in TAK through activation of the mTOR pathway in endothelial cells [34], promoting the progression of disease and leading to the lesion of cardiac valve eventually. Immunoglobulin is highly important for an effective immune response, produced by activated B cells and plasma cells in response to exposure to antigens [35]. We also found the infiltration of plasma cells in surgical specimen of aortic valve. Thus, considering higher level of inflammatory indicators such as ESR and hs-CRP in the aortic valve involvement group and inflammatory cells infiltrating in the resected aortic valve, immune disorder and inflammatory progression may be one of the important mechanisms of aortic valve lesion in TAK.

It is believed that dilatation of aortic root and ascending aorta were frequently observed in patients with TAK [36]. Dilation of the aortic root may be another mechanism of the high rate of aortic regurgitation in patients with TAK [5], which was confirmed in our research, showing higher proportion of dilated aortic root in patients with aortic valve involvement. We also identified the risk of aortic valve involvement in TAK patients with dilated aortic root diameter was 9.3-fold higher than in patients with normal aortic root diameter. Relative aortic insufficiency can occur when the aortic root diameter dilates to a certain extent. At the same time, we found lymphocytic infiltration of the aortic wall in 7 patients, with infiltration of plasma cells in 3 of them, which reflected the presence of active inflammation in the aortic root in TAK. The inflammation further damages the media of the aorta, leading to the rupture and disorder of elastic fibers in the media, which occurs in almost all the resected aortic wall in our research. The fragile aortic root, unable to bear the shear stress, becomes dilated [5] and then aortic valve regurgitation gradually develops. In our study, the risk of aortic valve involvement in TAK patients with dilated ascending aorta diameter was 4.5-fold higher than in patients with normal ascending aorta diameter.

Chronic AR can lead to left ventricular volume overload, which induces progressive left ventricular dilation and eccentric hypertrophy, ultimately leading to heart failure [37]. In our study, we found that people had larger LVEDD and left ventricular end-systolic diameter (LVESD) among TAK patients with aortic valvular lesion. LVEDD was reportedly as a major risk factor for long-term prognosis in TAK with the increased risk of combined end points by 3.6% once LVEDD increased by 1mm [37]. In addition, the level of BNP in TAK patients with aortic valve involvement was significantly higher. BNP is an excellent biomarker for heart failure, which levels increase in proportion to the severity of the heart failure [38]. Thus, TAK patients with aortic valve involvement should be treated actively to avoid further deterioration of cardiac function.

For TAK patients with severe AR and cardiac hemodynamic disorders, the surgical treatment of AR and control of inflammation may be the focus [5]. As for TAK patients with moderate-to-severe aortic regurgitation, Cheng reported that surgical treatment has a lower incidence of long-term events than conservative therapy by decreasing mortality and re-hospitalization [37], although the optimal surgical method has not been determined. As for thirty-four TAK patients who underwent aortic valve surgical treatment in our study, a total of 6 adverse events occurred during follow-up, including 5 in the isolated aortic valve surgery group and 1 in the Bentall group. A previous study about AR caused by aortitis showed that the patients after aortic root replacement had significantly decreased risk of cardiovascular death or reoperation compared with the patients after AVR [22]. In addition, control of inflammation plays an important role in the progression and prognosis of TAK [5], and aggressive postoperative control of inflammation is crucial to prevent complication such as prosthetic valve detachment and anastomotic aneurysm [21, 22]. A previous study found that active inflammation may be a predictor of pseudoaneurysm after surgical treatment of AR due to TAK [21]. The occurrence of anastomotic complications and remote cardiovascular events in TAK patients was found not to be related to the preoperative and/or postoperative use of steroids [21, 23, 39]. In our study, we found that preoperative anti-inflammatory therapy was not associated with prognosis, but patients who received regular anti-inflammatory therapy after surgery had a lower incidence of adverse events. Thus, aortic root replacement seems to be the optimal choice of surgical treatment in TAK patients with AR and regular anti-inflammatory therapy may reduce the occurrence of postoperative adverse events.

This study was a retrospective design and only a single-center trial with limited sample size. In the future, we hope to carry out multi-center and prospective research with a large sample size to get more persuasive statistical conclusions.

## Conclusions

In summary, this study showed that dilation of ascending aorta and aortic root, Numano type IIb, and elevated hs-CRP level are risk factors for patients developing aortic valve involvement in TAK. It is suggested that echocardiography should be performed routinely and termly to detect the lesion of aortic valve in TAK patients, and aggressive treatment should be performed to control inflammatory progression and avoid the further damage of aortic valve in the early stages of the disease. As for TAK patients with aortic valve surgery, aortic root replacement seems to be the preferred option and regular anti-inflammatory therapy may reduce the occurrence of adverse events of them.

## Supplementary Information

**Additional file 1: Supplementary Table 1.** Medication of TAK patients with or without aortic valve involvement before first admission. **Supplementary Table 2.** Laboratory tests and disease activity scores of TAK patients with or without aortic valve involvement. **Supplementary Table 3.** Predictive factors of adverse events in TAK patients with surgical treatment for aortic valve lesion.

## Data Availability

The datasets used and/or analyzed during the current study are available from the corresponding author on reasonable request.

## Abbreviations

TAK: Takayasu arteritis
hs-CRP: High-sensitivity C-reactive protein
OR: Odds ratio
CI: Confidence interval
HR: Hazard ratio
AR: Aortic regurgitation
ITAS: Indian Takayasu’s Arteritis Activity Score
ITAS-A: ITAS with acute-phase reactants
ESR: Erythrocyte sedimentation rate
TNF-α: Tumor necrosis factor alpha
IL: Interleukin
Ig: Immunoglobulin
EF: Ejection fraction
LVEDD: Left ventricular end-diastolic diameter
BNP: Brain natriuretic peptide
cTnI: Cardiac troponin I
WBC: White blood cell
RBC: Red blood cell
AVR: Aortic valve replacement
LVESD: Left ventricular end-systolic diameter
