# Supplementary material for: Risk factors and surgical prognosis in patients with aortic valve involvement caused by Takayasu arteritis
Source: Arthritis Res Ther. 2022 May 7;24:102. doi: 10.1186/s13075-022-02788-9 (PMC9077813; doi:10.1186/s13075-022-02788-9)
Supplement: Supplementary file 1 — Additional file 1: Supplementary Table 1. Medication of TAK patients with or without aortic valve involvement before first admission. Supplementary Table 2. Laboratory tests and disease activity scores of TAK patients with or without aortic valve involvement. Supplementary Table 3. Predictive factors of adverse events in TAK patients with surgical treatment for aortic valve lesion. [file 13075_2022_2788_MOESM1_ESM.docx]

Supplementary Table1. Medication of TAK patients with or without aortic valve involvement before first admission.

| Variable | Total  (n=172) | Aortic valve involvement  (n=92) | Without aortic valve involvement  (n=80) | P |
| --- | --- | --- | --- | --- |
| Glucocorticoid, n (%) | 53（31.5） | 32（36.4） | 21（26.3） | 0.159 |
| Cyclophosphamide, n (%) | 25（14.9） | 14（15.9） | 11（13.8） | 0.695 |
| Methotrexate, n (%) | 10（6.0） | 5（5.7） | 5（4.8） | 1.000 |
| Azathioprine, n (%) | 4（2.4） | 3（3.4） | 1（1.3） | 0.682 |
| Mycophenolate Mofetil, n (%) | 9（5.4） | 6（6.8） | 3（3.8） | 0.590 |
| Hydroxychloroquine, n (%) | 6（3.6） | 3（3.4） | 3（3.8） | 1.000 |
| Cyclosporin A, n (%) | 1（0.6） | 1（1.1） | 0（0.0） | 1.000 |
| Leflunomide, n (%) | 4（2.4） | 3（3.4） | 1（1.3） | 0.682 |
| Tripterygium wilfordii, n (%) | 2（1.2） | 1（1.1） | 1（1.3） | 1.000 |
| Tacrolimus, n (%) | 1（0.6） | 1（1.1） | 0（0.0） | 1.000 |
| Tocilizumab, n (%) | 3（1.8） | 1（1.1） | 2（2.5） | 0.934 |

Supplementary Table2. Laboratory tests and disease activity scores of TAK patients with or without aortic valve involvement.

| Variable | Total  (n=172) | Aortic valve involvement  (n=92) | Without aortic valve involvement  (n=80) | P |
| --- | --- | --- | --- | --- |
| WBC，10^9^/L | 6.96（5.62,8.33） | 7.10（5.86,8.19） | 6.73（5.49,8.78） | 0.738 |
| RBC，10^12^/L | 4.29(3.99,4.65) | 4.24(3.97,4.54) | 4.37(4.00,4.75) | 0.062 |
| Platelet，10^9^/L | 251.50  (198.00,308.75) | 244.00  (192.00,305.00) | 266.50  (211.75,323.25) | 0.064 |
| ALT，U/L | 13.00  （9.00,21.00） | 12.00  （9.00,20.00） | 13.00  （10.00,22.00） | 0.342 |
| Creatinine，umol/l | 56.55  （47.98,65.93） | 57.40  （49.20,65.50） | 54. 00  （46.70,66.60） | 0.383 |
| Elevated ESR, mm/h | 65(38.5) | 41(45.6) | 24(30.4) | 0.043 |
| Elevated hs-CRP, g/l | 81(49.1) | 53(60.2) | 28(36.4) | 0.002 |
| Elevated TNF-α, n(%) | 79（71.8） | 31（67.4） | 48（75.0） | 0.382 |
| Elevated IL-6, n (%) | 52（46.8） | 23（48.9） | 29（45.3） | 0.705 |
| Elevated IgA, n (%) | 15（10.3） | 9（13.2） | 6（7.7） | 0.271 |
| Elevated IgG, n (%) | 26（17.8） | 18（26.5） | 8（10.3） | 0.011 |
| Elevated IgM, n (%) | 13（8.9） | 7（10.3） | 6（7.7） | 0.582 |
| Elevated C3, n (%) | 13（8.8） | 4（5.7） | 9（11.5） | 0.211 |
| Elevated C4, n (%) | 10（7.0） | 4（6.1） | 6（7.9） | 0.923 |
| BNP，pg/ml | 106.00  (31.50,359.50) | 189.00  (50.00,434.00) | 34.50  （25.25,145.25） | 0.004 |
| cTnI，ng/ml | 0.00（0.00,0.02） | 0.01（0.00,0.19） | 0.00（0.00,0.00） | 0.001 |
| NIH score | 2.00(2.00,3.00) | 2.00(2.00,3.00) | 2.00(2.00,3.00) | 0.055 |
| ITAS2010 score | 6.00  (3.50,9.00) | 7.00(4.75,9.00) | 5.00(3.00,9.00) | 0.051 |
| ITAS-A score | 8.00  (5.00,11.00) | 9.00  (6.75,13.00) | 6.00  (4.00,10.00) | ＜0.001 |

Abbreviations: WBC, white blood cell; RBC, red blood cell; ALT, alanine transaminase; ESR, erythrocyte sedimentation rate; hs-CRP, high-sensitivity C-reactive protein; TNF, tumor necrosis factor; IL, interleukin; Ig, immunoglobulin; C3, complement 3; C4, complement 4; BNP, brain natriuretic peptide; cTnI, cardiac troponin I.

Supplementary Table3. Predictive factors of adverse events in TAK patients with surgical treatment for aortic valve lesion.

| Variable | P-valve | Hazard ratio(95%CI) |
| --- | --- | --- |
| Age at symptom onset, years | 0.310 | 0.968(0.909,1.031) |
| Duration of disease, months | 0.303 | 1.005(0.996,1.013) |
| Surgical methods | 0.024 | 0.082(0.01,0.715) |
| Elevated ESR, mm/h | 0.757 | 0.765(0.140,4.179) |
| Elevated hs-CRP, mg/l | 0.418 | 0.478(0.080,2.859) |
| EF，% | 0.154 | 1.079(0.972,1.198) |
| LVEDD，mm | 0.172 | 0.931(0.840,1.032) |
| LAD＞38mm | 0.249 | 3.594(0.409,31.577) |
| Ascending aorta diameter, mm | 0.569 | 1.023(0.946,1.106) |
| Aortic root diameter, mm | 0.579 | 0.965(0.851,1.094) |
| Preoperatively anti-inflammatory therapy | 0.126 | 0.261(0.047,1.462) |
| Glucocorticoid | 0.200 | 0.321(0.056,1.828) |
| Immunosuppressant/Biological agents | 0.488 | 0.467(0.054,4.027) |
| Postoperatively anti-inflammatory therapy | 0.036 | 0.144(0.023,0.881) |
| Glucocorticoid | 0.249 | 0.366(0.066,2.019) |
| Immunosuppressant/Biological agents | 0.437 | 2.030(0.340,12.133) |

Abbreviations: ESR, erythrocyte sedimentation rate; hs-CRP, high-sensitivity C-reactive protein; EF%, cardiac ejection fraction; LVEDD, left ventricular end-diastolic diameter; LAD, left atrial diameter.
